# Evaluation of the Results of a Multicomponent Emotional Health Intervention in a School Setting

**DOI:** 10.3390/ejihpe16070102

**Published:** 2026-07-15

**Authors:** Eva-María Barroso-Márquez, María-de-los-Ángeles Merino-Godoy, Izaro Eraña-Méndez, David Gómez-Asencio, Yeray Cabrera-Arana, Cristina Arana-Álvarez, Francisco-Javier Gago-Valiente, Eva-María Carrasco-Barroso

**Affiliations:** 1 Department of Nursing, Faculty of Nursing, University of Huelva, 21071 Huelva, Spain; eva.barroso@denf.uhu.es (E.-M.B.-M.); angeles.merino@denf.uhu.es (M.-d.-l.-Á.M.-G.); izaro.erana@alu.uhu.es (I.E.-M.); 2 Research Centre on Contemporary Thought and Innovation for Social Development (COIDESO), University of Huelva, 21071 Huelva, Spain; evamaria.carrasco@alu.uhu.es; 3 Department of Pedagogy, Faculty of Education, Psychology and Sport Sciences, University of Huelva, 21071 Huelva, Spain; david.gomez419@alu.uhu.es (D.G.-A.); yeray.cabrera@alu.uhu.es (Y.C.-A.); cristina.arana530@alu.uhu.es (C.A.-Á.)

**Keywords:** emotional health, adolescence, anxiety disorders, social phobia, school intervention program, RCADS, internalizing comorbidity, sex differences, school nursing

## Abstract

Background: The global prevalence of anxiety and internalizing disorders in adolescence has increased epidemiologically over the past decade, compounded by the impact of the COVID-19 pandemic and the expansion of digital social networks. Despite the availability of empirically based interventions, their systematic implementation in Spanish educational settings—especially in socioeconomically vulnerable environments—remains insufficient. This study aimed to assess the emotional symptomatology profile in a sample of adolescents enrolled in the 2nd year of Compulsory Secondary Education (CSE) and to describe the changes in symptomatology observed after implementation of a multicomponent intervention program in an ordinary school setting. Methods: a quasi-experimental single-group repeated-measures design was used, with pretest–posttest measurements (T1–T2). The sample included 79 participants (age in completed years: M = 13.28 years, SD = 0.64; range: 12–15; 49.4% female, 44.3% male, 6.3% undisclosed), selected by convenience at IES Estuaria (Huelva). The assessment instrument was the Revised Child Anxiety and Depression Scale (RCADS-47), which measures total anxiety, total internalization, and six clinical subdimensions aligned with DSM-5: generalized anxiety disorder (GAD), major depressive disorder (MDD), panic disorder (PD), separation anxiety (SA), social phobia (SP), and obsessive–compulsive disorder (OCD). The intervention consisted of five multicomponent group workshops (45 min/session; three class groups) integrating emotional psychoeducation, receptive music therapy, cognitive–behavioral emotional regulation, assertive communication, and body expression/mindfulness. Results: At pretest, Social Phobia (SP) showed the highest clinical impairment, significantly affecting more females (66.67%) than males (20.00%). Generalized Anxiety Disorder (GAD) also presented high impairment in females. Significant correlations between pairs of subdimensions revealed a high comorbidity pattern. Following the intervention, reductions in clinically significant levels were observed across all dimensions. Matched-pairs Wilcoxon signed-rank tests confirmed statistically significant score reductions from T1 to T2 in Total Anxiety, Total Internalization, Social Phobia, and Generalized Anxiety Disorder (all *p* < 0.05), with the largest effect for Social Phobia (r = 0.46); no significant change was detected for Panic Disorder, Separation Anxiety, Major Depressive Disorder, or Obsessive–Compulsive Disorder, although the direction of change favored improvement in all cases. Conclusions: The multicomponent intervention program produced improvements in emotional symptomatology over the short-term follow-up period, although without achieving complete clinical remission in any subdimension. The persistent sex differences—with greater residual impairment in the female group after intervention—underscore the need to incorporate a gender perspective in the design of school emotional health programs. High comorbidity rates between subdimensions support the utility of transdiagnostic approaches. Implications for school nursing practice and future research are discussed.

## 1. Introduction

Adolescence constitutes a critical period of human development in which the confluence of biological, cognitive, and socioaffective transformations creates optimal conditions for the emergence of internalizing psychopathology (Kessler et al., 2005; Merikangas et al., 2010). From a neurobiological perspective, adolescence is characterized by asynchronous maturation between the limbic system—which reaches functional peak at an early age—and the prefrontal cortex—whose myelination is not complete until approximately age 25—, generating a vulnerability window in which emotional reactivity exceeds cognitive regulation capacity (Casey et al., 2008; Steinberg, 2008). This vulnerability is epidemiologically expressed in concerning prevalence rates: according to the World Health Organization (WHO, 2022), approximately one in seven adolescents globally meets criteria for a diagnosable mental disorder, with anxiety disorders being the most frequent (lifetime prevalence ≈ 32%; Merikangas et al., 2010) and earliest in onset (mean age of onset: 11 years). In Spain, the HBSC-2022 National Health Survey report documented a significant and sustained deterioration in emotional well-being among the adolescent population over the last decade, with a disproportionate increase in females (Moreno et al., 2025); a trend that was accelerated by the COVID-19 pandemic (Ma et al., 2021; Racine et al., 2021).

Internalizing disorders refer to a group of psychological conditions characterized by innerdirected distress, such as anxiety, depression, and somatic complaints, where individuals process psychological stress inwardly rather than expressing it through disruptive external behaviors. These anxiety and internalizing disorders in adolescence are not isolated entities, but rather organize into a high-comorbidity psychopathological spectrum mediated by shared vulnerability factors—neuroticism, behavioral inhibition, and emotional dysregulation—which Krueger and Markon (2006) conceptualized under the so-called ‘p’ factor of general psychopathology. Emotional regulation—defined as the set of extrinsic and intrinsic processes responsible for monitoring, evaluating, and modifying emotional reactions (Thompson, 1994; Gross, 2015)—occupies the central place in this model, as maladaptive regulation strategies (rumination, suppression, avoidance) act as transdiagnostic mediators between perceived stress and the clinical expression of internalizing symptomatology (Aldao et al., 2010; Compas et al., 2017). This has direct implications for intervention design: the most effective school programs are those that address these common mechanisms—rather than specific isolated disorders—adopting a transdiagnostic perspective (Barlow et al., 2017).

The contemporary sociotechnological context has introduced new risk vectors that amplify adolescent vulnerability. The massive use of digital social communication platforms has established an omnipresent and quantified social evaluation ecosystem (likes, comments, followers) that exacerbates upward social comparison mechanisms and body objectification, which is particularly deleterious for social anxiety in adolescent females (Fardouly & Vartanian, 2015; Keles et al., 2020). In a meta-analysis of 13 longitudinal studies, Coyne et al. (2020) documented that problematic social media use is associated with increases in anxiety and depression symptoms in adolescents aged 13 to 18, with greater effect sizes in females. This reality was worsened by the COVID-19 pandemic, which imposed conditions of prolonged social isolation and increased dependence on digital interaction, with documented adverse effects on anxiety, depression, and social functioning in adolescent samples across Europe and Latin America (Racine et al., 2021; Quero et al., 2021).

Against this backdrop, the educational environment emerges as the preventive intervention space with the greatest potential for population impact, as it enables universal access to adolescents at the developmental moment of greatest plasticity and vulnerability (Weare & Nind, 2011; Durlak et al., 2011). Meta-analyses on school-based Social-Emotional Learning (SEL) programs document significant effects on the development of emotional competencies, the reduction in internalizing symptoms, and the improvement of academic performance (Durlak et al., 2011; average d = 0.66 in competencies; d = 0.24 in internalizing symptoms). Multicomponent programs that integrate music therapy techniques, cognitive–behavioral emotional regulation skill training, assertive communication, and mind–body interventions have shown specific favorable effects on social anxiety and generalized anxiety in adolescents (Thaut & Hoemberg, 2014; Sibinga et al., 2016; Mehling et al., 2011), although evidence in Spanish educational settings remains limited.

Sex differences in the presentation and course of internalizing disorders constitute one of the most robust and replicated findings in adolescent mental health epidemiology. Adolescent females present prevalence rates of anxiety and mood disorders between 1.5 and 3 times higher than their male peers, a gap that consistently emerges from ages 12–13 and is maintained throughout adulthood (McLean et al., 2011; Hankin et al., 1998). This disparity is not reducible to a single cause, but results from the interaction of three levels of determination: (a) biological, including greater reactivity of the hypothalamic–pituitary–adrenal (HPA) axis to social stressors mediated by estrogens, and greater amygdala activation to threatening stimuli observed in functional neuroimaging (Guyer et al., 2008; Goel & Bale, 2010); (b) cognitive, including females’ greater tendency toward rumination, catastrophizing, and sensitivity to negative social evaluation (Nolen-Hoeksema & Hilt, 2009); and (c) sociocultural, including adolescent girls’ greater exposure to body objectification and appearance standards disseminated by digital media (Fredrickson & Roberts, 1997; Fardouly & Vartanian, 2015). These mechanisms have direct implications for designing interventions that aim to reduce the gender gap in emotional health.

Based on this theoretical and empirical framework, this study set out the following specific objectives: (O1) to characterize the emotional profile in a sample of 2nd-year CSE adolescents using the RCADS; (O2) to analyze the association between RCADS scores and the sex variable; (O3) to explore the statistical dependency relationships between clinical RCADS subdimensions; (O4) to examine the correlations between RCADS scores and age; and (O5) to evaluate changes in symptomatology after implementation of a multicomponent intervention program. The main hypothesis states that the program will be associated with a reduction in RCADS impairment levels at posttest compared to pretest, with differences in magnitude depending on sex; given the descriptive nature of the pre–post analysis reported here, this hypothesis is addressed at a descriptive level and should be confirmed through inferential testing in future controlled studies.

## 2. Materials and Methods

### 2.1. Study Design

The study employed a quasi-experimental single-group design with repeated measures at two time points—pretest (T1) and posttest (T2)—following the methodological recommendations of Shadish et al. (2002) for intervention evaluation studies in natural settings. This design, preferred over randomized experimental designs when randomization is not feasible for ethical, logistical, or school group integrity reasons, allows quantification of intragroup changes attributable to the intervention, although its main limitation lies in the impossibility of fully controlling concurrent confounding factors (history, maturation, testing effects). The methodology is framed within the quantitative paradigm of a descriptive-correlational nature with an intervention evaluation phase (Campbell & Stanley, 1963; Blaxter et al., 2008). The study is reported following the TREND guidelines (Des Jarlais et al., 2004) for non-randomized behavioral and public health intervention studies.

### 2.2. Setting and Participants

The study was conducted at IES Estuaria in Huelva, a bilingual public secondary school founded in 1983 and located in the Isla Chica neighborhood—one of the most populous in the municipality, with 19,406 inhabitants, predominantly Spanish composition (91.5%), and a predominantly working-class socioeconomic profile linked to the petrochemical industrial sector and self-employed trade (Ayuntamiento de Huelva, 2024). The school offers exclusively CSE, with twelve class groups distributed across four levels and three streams (A, B, C), and is enrolled in the ‘Forma Joven’ program of the Junta de Andalucía (Ayuntamiento de Huelva, 2024). The target population consisted of all students enrolled in the three 2nd-year CSE groups for the 2025–2026 academic year (approximate n = 90), a level identified by the school counselor as having the greatest need for emotional health intervention based on prior systematic observations. These observations included concerns regarding gender-based social dynamics and school climate within this student cohort, as reported by the school counselor and class tutors.

### 2.3. Eligibility Criteria and Sample

Inclusion criteria were (a) enrollment in 2nd-year CSE at IES Estuaria in the 2025–2026 academic year; (b) written informed consent from the legal guardian; and (c) voluntary willingness to participate. Exclusion criteria were (a) not being enrolled in 2nd-year CSE at IES Estuaria; (b) absence of legal consent; and (c) refusal to participate. No exclusion criteria were established based on prior diagnosis or academic performance. The final sample consisted of n = 79 participants (participation rate among students with authorized consent: 100%). Five participants (6.3%) were absent at the T2 assessment session, yielding an analytic sample of n = 74 at posttest. This figure reflects absence from the T2 assessment session specifically; attendance across the five intervention sessions was not tracked at the individual level (see Section 2.6).

### 2.4. Measurement Instruments

Sociodemographic variables collected were (i) age, in completed years (self-reported discrete quantitative variable); (ii) sex, a self-reported polytomous nominal qualitative variable (categories: female, male, prefer not to say); and (iii) cultural background (polytomous nominal qualitative variable). The main assessment instrument was the Revised Child Anxiety and Depression Scale (RCADS-47; Chorpita & Spence, 1998, 2018). The RCADS is a 47-item self-report scale designed for the assessment of anxiety and depression symptoms in children and adolescents aged 8 to 18, developed from DSM-IV diagnostic criteria and widely used in both clinical research and educational settings. Items are answered on a 4-point Likert scale (0 = Never; 1 = Sometimes; 2 = Often; 3 = Always). The psychometric properties of the instrument are robust: internal consistency Cronbach’s α = 0.70–0.96 depending on the subscale; test–retest reliability ICC = 0.76–0.86; adequate convergent validity (correlations r = 0.55–0.82 with the Multidimensional Anxiety Scale for Children [MASC] and the Children’s Depression Inventory [CDI]); and discriminant validity documented in clinical and community samples (Chorpita et al., 2005; de Ross et al., 2002). The Spanish version used was the RCADS47-Youth-Spanish (Chorpita & Spence, 2018). The instrument generates scores on two higher-order dimensions—total anxiety and total internalization—and six specific subdimensions: GAD (6 items; α = 0.74), MDD (10 items; α = 0.87), PD (9 items; α = 0.89), SA (7 items; α = 0.79), SP (9 items; α = 0.82), and OCD (6 items; α = 0.72). Scores are interpreted through normative percentiles that classify participants into four impairment levels: normal, mild impairment, clinically significant, and severe impairment.

### 2.5. Intervention Program

The intervention program was designed and implemented by a multidisciplinary team comprising a clinical psychologist, an early childhood education teacher, a music education specialist with music therapy training, research staff from the University of Huelva, and a nursing graduate with high-performance sport training and somatic mindfulness training. Program design followed the principles of evidence-based psychosocial intervention planning (Bartholomew et al., 2016) and was grounded in the literature on effective SEL programs (Durlak et al., 2011; Weare & Nind, 2011). The program included five group workshops of 45 min each, implemented across three class groups (n = 3 groups; 12 total sessions; 4 sessions per group) in person. The active components of the intervention were (a) emotional psychoeducation (Session 1), aimed at increasing emotional literacy and intrinsic motivation; (b) receptive and active music therapy (Session 2), employing musical stimuli as a facilitator of emotional identification and expression (Thaut & Hoemberg, 2014); (c) cognitive–behavioral emotional regulation (Session 3), incorporating the cognitive-emotional cycle model, cognitive restructuring, and cognitive defusion from Acceptance and Commitment Therapy (ACT; Hayes et al., 2006); (d) assertive communication and social skills training (Session 4), based on Alberti and Emmons’ (1978) tripartite model and forum theater as an active methodology; and (e) body expression and somatic mindfulness (Session 5), supported by evidence on downward autonomic regulation through mind–body interventions (Mehling et al., 2011; Sibinga et al., 2016; Röhricht, 2009). The detailed content of each session is described in Table 1.

### 2.6. Data Collection Procedure

The data collection protocol was structured into four sequential phases: (1) institutional coordination, through a meeting with the management team—school counselor, vice-principal, and class group tutors—to agree on objectives, schedule, and implementation conditions; (2) ethical approval and consent, with a request to the Huelva Research Ethics Committee (code SICEIA-2025-003511; study code TFG-SEPAUIE-2025) and distribution of information sheets and written informed consent forms to the legal guardians of participants; (3) pretest administration (T1), conducted in three consecutive teaching sessions, with permanent presence of the class tutor and at least one researcher to ensure understanding of instructions and correct completion of the scale; and (4) posttest administration (T2), at the closing session of each group’s intervention, under conditions identical to T1. As the study’s primary focus was the evaluation of pre-post change in emotional symptomatology, the data collection protocol prioritized supervised administration of the pretest (T1) and posttest (T2) assessments; individualized attendance at the intervention sessions themselves was not systematically recorded.

### 2.7. Statistical Analysis

Statistical analysis was performed using IBM SPSS Statistics version 25.0 (IBM Corp., Armonk, NY, USA). The statistical significance level was set at α = 0.05 (two-tailed tests) for all analyses. A post hoc power analysis was conducted with G*Power 3.1 (Faul et al., 2007) to estimate the statistical power available for the main inferential tests given the obtained sample size (n = 79).

Descriptive analyses included: for quantitative variables, the arithmetic mean (M), standard deviation (SD), median (Mdn), interquartile range (IQR), and extreme values; for categorical variables, absolute frequencies (n) and percentages (%). The univariate normality assumption was tested using the Kolmogorov–Smirnov test with Lilliefors correction (D), selected as most appropriate when n > 50 (Field, 2018); variables with *p* < 0.05 were treated as non-normal. Table 2 summarizes the normality test results for all quantitative study variables. Age and the SA, MDD, PD, and OCD subdimensions did not meet the normality assumption (D between 0.128 and 0.440; *p* < 0.05), while GAD, SP, total anxiety, and total internalization did (D between 0.058 and 0.091; *p* ≥ 0.177). Accordingly, Spearman’s Rho was used for correlations involving non-normally distributed variables, and Pearson’s r for normally distributed variable pairs.

For bivariate cross-sectional analyses, the association between categorical variables was examined with Pearson’s Chi-square test (χ^2^), supplemented with Yates’ correction when any expected frequency was less than 5; effect size was estimated using Cramer’s V (small ≥ 0.10; medium ≥ 0.30; large ≥ 0.50; Cohen, 1988). For correlation analyses, Spearman’s Rho (ρ) was used when any of the involved variables did not meet the normality assumption, and Pearson’s r coefficient was used for normally distributed variable pairs. Post hoc power analysis for χ^2^ tests (with df = 4; n = 79) indicated 1 − β = 0.74 for a medium effect (V = 0.30) and 1 − β = 0.42 for a small effect (V = 0.10), confirming adequate power to detect moderate associations and limited power for small effects.

Pre-post intervention changes (T1 → T2) are described as absolute differences in percentage points (Δ, pp) within each impairment category, calculated separately for each sex group, to characterize shifts in the distribution of severity levels. In addition, inferential pre-post comparisons were conducted on matched pairs using the Wilcoxon signed-rank test for related samples, applied to the continuous subscale and total scores of each participant at T1 and T2; results were corroborated with a two-tailed sign test. The non-parametric approach was retained uniformly across dimensions—rather than a paired-samples *t*-test for the subscales meeting the normality assumption (Table 2)—because the normality of the raw scores does not guarantee the normality of the T1–T2 difference scores, and a single consistent procedure was preferred for comparability across dimensions. Effect sizes are reported as the matched-pairs rank-biserial correlation (r), interpreted following Cohen’s (1988) conventions (small ≥ 0.10; medium ≥ 0.30; large ≥ 0.50).

### 2.8. Ethical Considerations

The study received approval from the Research Ethics Committee (REC) of Huelva (code SICEIA-2025-003511; study code TFG-SEPAUIE-2025). The design and conduct of the study fully adhered to the principles of the Declaration of Helsinki (WMA, 2013), the Code of Good Scientific Practices CSIC (2017), and current data protection regulations: Regulation (EU) 2016/679 (GDPR) and Organic Law 3/2018 on Personal Data Protection and Guarantee of Digital Rights. Since all participants were minors, written informed consent was provided by their legal guardians, following receipt of an information sheet describing the objectives, procedures, possible risks and benefits, confidentiality, and the voluntary and revocable nature of participation. No financial or academic compensation was planned for participation. Program activities were institutionally framed within the Junta de Andalucía’s ‘Forma Joven’ program, covered by LOMLOE and Law 41/2002 regulating patient autonomy. The research did not involve clinical interventions, drug administration, or biological sample collection. Data security was ensured through pseudonymization, encrypted storage, and restricted access limited to the research team.

## 3. Results

### 3.1. Pretest Evaluation (T1)

#### 3.1.1. Sociodemographic Characteristics of the Sample

The sample consisted of 79 participants with a mean age of M = 13.28 years, measured in completed years via self-report (SD = 0.64; Mdn = 13; IQR = 1; range: 12–15). Sex distribution was 49.4% female (n = 39), 44.3% male (n = 35), and 6.3% preferred not to disclose (n = 5). Cultural background was predominantly Spanish across all groups (>90%), consistent with the neighborhood’s demographic composition (Ayuntamiento de Huelva, 2024). No missing data were recorded for sociodemographic variables.

#### 3.1.2. Dimensions of the RCADS

Table 3 presents the consolidated distribution of RCADS dimensions and subdimensions by sex at pretest (T1) and posttest (T2). Data for the subgroup that preferred not to disclose their sex (n = 5 at T1; n = 4 at T2) are included for consistency with the rest of the analysis; however, given the small subgroup size, these values do not allow robust statistical interpretation and should be regarded as purely descriptive. In the higher-order dimensions, most participants fell within the normative range; however, sex differences were substantial and consistent (Table 3). For total anxiety, 30.77% of females reached the clinically significant threshold, compared to 11.43% of males (absolute difference: 19.34 pp). For total internalization, this divergence was equally marked: 25.64% versus 5.71% (absolute difference: 19.93 pp). The undisclosed subgroup showed a profile distributed within normal and mild impairment ranges, with no clinically significant cases, although the small subgroup size (n = 5) limits interpretation of these data.

#### 3.1.3. Subdimensions of the RCADS

Analysis at the level of clinical subdimensions revealed a heterogeneous pattern (Table 3). SA was the subdimension with the lowest overall prevalence of clinical impairment, with more than 79% of participants within the normal range regardless of sex. At the other extreme, SP and GAD showed the highest impairment profiles. For SP, 66.67% of females reached the clinically significant level—the highest percentage across all subdimensions and groups—in contrast to 20.00% of males. For GAD, only 17.95% of females fell in the normal range, with 46.15% accumulating across the clinically significant and severe levels. For MDD, PD, and OCD, the female group also showed higher impairment at upper levels. Without exception across all subdimensions, the female sex showed impairment percentages equal to or greater than the male group.

### 3.2. Bivariate Analyses

#### 3.2.1. Association Between Sex and RCADS Dimensions

The Chi-square test between sex and the two higher-order dimensions did not reach statistical significance (total anxiety: χ^2^ = 8.231, df = 4, *p* = 0.083; total internalization: χ^2^ = 7.645, df = 4, *p* = 0.106), although effect sizes (V = 0.229 and V = 0.220, respectively) approached the conventional small-to-moderate effect threshold (Cohen, 1988), suggesting that the reduced sample size may have compromised statistical power to detect these differences. At the subdimension level, only SP showed a statistically significant association with sex (χ^2^ = 16.455, df = 4, *p* = 0.002; Cramer’s V = 0.323), an effect size that exceeds the moderate effect threshold and is clinically relevant. A total of 66.67% of female participants were at the clinically significant level, compared to 20.00% of males. For the remaining subdimensions, the Chi-square test did not reach statistical significance (GAD: *p* = 0.091; MDD: *p* = 0.283; PD: *p* = 0.136; SA: *p* = 0.468; OCD: *p* = 0.071).

#### 3.2.2. Associations Between RCADS Subdimensions

Systematic analysis of the dependency relationships between all pairs of RCADS subdimensions using Pearson’s Chi-square test revealed 12 statistically significant associations out of a total of 15 possible pairs (Table 4). *p*-values ranged from <0.001 (MDD ↔ SA and PD ↔ OCD; both df = 6 and df = 9, respectively) to 0.034 (SA ↔ SP; df = 4). Effect sizes, however, showed that the strongest associations corresponded to the MDD-SA (V = 0.753) and PD-OCD (V = 0.612) pairs, both classified as large, while SA-SP, PD-SP, and SP-OCD showed moderate effect sizes (V = 0.257–0.324) despite reaching statistical significance. Non-significant pairs were GAD ↔ SA, GAD ↔ MDD, and MDD ↔ SP. This pattern of interdependencies is theoretically consistent with the internalizing spectrum model (Krueger, 1999; Krueger & Markon, 2006) and suggests high comorbidity burden in this sample.

#### 3.2.3. Correlations Between RCADS Subdimensions and Age

As established in Section 2.7 (Table 2), age and the SA, MDD, PD, and OCD subdimensions did not meet the normality assumption, whereas GAD, SP, total anxiety, and total internalization did; consequently, Spearman’s Rho was systematically employed for correlations involving age. Spearman Rho correlations between age and subdimensions SA, MDD, and PD were negative and did not reach statistical significance (ρ between −0.026 and −0.197; *p* between 0.081 and 0.822), indicating no monotonic linear relationship between age and these dimensions in the age range studied (Table 5). Correlations between subdimensions themselves were substantially higher: the MDD-PD pair showed the highest coefficient (ρ = 0.681, *p* < 0.001), followed by SA-PD (ρ = 0.579, *p* < 0.001) and SA-MDD (ρ = 0.411, *p* < 0.001), corroborating the internalizing comorbidity pattern documented in the Chi-square analysis.

In the analysis of normally distributed variables (SP, GAD, total anxiety, and total internalization), all inter-subdimension correlations were positive, strong, and highly significant (ρ between 0.587 and 0.979; *p* < 0.001), with the near-perfect relationship between total anxiety and total internalization standing out (ρ = 0.979, expected given their overlapping composition). Pearson’s r correlation between age and SP was negative and statistically significant (r = −0.270, *p* = 0.016), indicating a moderate reduction in social anxiety with advancing age in this developmental period. Correlations of age with GAD (r = 0.015), total anxiety (r = −0.119), and total internalization (r = −0.100) did not reach statistical significance (Table 6).

### 3.3. Posttest Evaluation (T2): Changes Following Intervention

Following implementation of the multicomponent intervention program, a generalized improvement trend was recorded across all evaluated RCADS dimensions and subdimensions (Table 3). The magnitude of observed changes is expressed as variation in percentage points (Δ, pp) in the proportion of participants at the clinically significant and normal levels, comparing T2 with T1.

For SP, the reduction in the clinically significant level was the most pronounced across all dimensions: Δ = −25.0 pp in the female group (from 66.67% to 41.7%) and Δ = −14.1 pp in the male group (from 20.0% to 5.9%). In parallel, normal levels increased by Δ = +15.0 pp (females) and Δ = +27.2 pp (males). For GAD, the female group showed a reduction of 21.8 pp in the clinically significant category (from 38.46% to 16.7%) and an increase of 15.4 pp in the normal category (from 17.95% to 33.3%); in males, the reduction was 11.0 pp (from 25.71% to 14.7%). For PD, the proportion at the normal level increased by Δ = +10.5 pp for females and Δ = +19.6 pp for males. For total anxiety, normal levels increased by Δ = +17.9 pp in females and Δ = +14.1 pp in males. For total internalization, the reduction in the clinically significant level was Δ = −9.0 pp in females and Δ = +0.4 pp in males (slight change). The undisclosed subgroup showed a trend toward the normal range in total internalization (Δ = +40.0 pp), although the extreme small size of this subgroup (n = 4 at T2) prevents any statistical interpretation of this result. It should be noted that a ‘severe impairment’ category emerged in SP at T2 that was not present at T1 (8.3% in females; 2.9% in males), a finding that requires interpretive caution as it could reflect a measurement artifact or a sensitization effect to the symptomatology itself induced by the program.

Inferential testing on matched pairs (Wilcoxon signed-rank test; Table 7) confirmed statistically significant score reductions from T1 to T2 in Total Anxiety (Z = −2.86, *p* = 0.004, r = 0.34), Total Internalization (Z = −2.85, *p* = 0.004, r = 0.33), Social Phobia (Z = −3.93, *p* < 0.001, r = 0.46), and Generalized Anxiety Disorder (Z = −2.08, *p* = 0.037, r = 0.24), with the largest effect observed for Social Phobia. No statistically significant pre–post change was detected for Panic Disorder, Separation Anxiety, Major Depressive Disorder, or Obsessive–Compulsive Disorder (all *p* > 0.05), although the direction of change favored improvement in each case (more participants improved than worsened; Table 7). These results were corroborated by a two-tailed sign test, which yielded convergent significance patterns across all eight dimensions.

## 4. Discussion

This study examined the emotional profile in a sample of 2nd-year CSE adolescents in a socioeconomically disadvantaged setting, and described the changes in symptomatology observed following implementation of a multicomponent intervention program in an ordinary school setting. The results obtained are discussed in relation to four thematic axes: (1) the baseline symptomatology profile and its epidemiological context; (2) sex differences and their determinants; (3) internalizing comorbidity patterns and their transdiagnostic implications; and (4) observed changes following the intervention and possible mechanisms of action.

The high prevalence of clinically significant social phobia documented at baseline—44.3% of the total sample; 66.67% in the female group—substantially exceeds the point prevalence estimates reported in Spanish normative studies (point prevalence of SP in CSE adolescents ≈ 4–7%; Ortuño-Sierra et al., 2017) and approaches the prevalences reported in clinical samples (≈50–60%; Rao et al., 2007). This discrepancy can be explained by three non-exclusive factors: (a) the use of the RCADS as a screening tool—which tends to overestimate prevalence compared to structured diagnostic interviews—; (b) evaluation conditions—a group classroom context, which may induce greater recognition of social symptomatology—; and (c) a genuine post-pandemic increase in social anxiety documented in several European populations (Ma et al., 2021; Racine et al., 2021). The amplifying effect of digital hyperconnectivity on social evaluation—a mechanism specifically documented in Spanish adolescents (Fardouly & Vartanian, 2015; Keles et al., 2020)—should also be considered, although the non-experimental nature of the design prevents establishing causal inferences. In addition to these methodological considerations, school staff (counselor and class tutors) had independently reported, prior to the intervention, gender-based social dynamics and school climate concerns within this cohort (Section 2.2); while based on informal, non-validated observations rather than a diagnostic instrument, this contextual information is consistent with the markedly elevated baseline SP prevalence observed among females and may partly explain it. The high prevalence of GAD (46.15% of females with clinically significant or severe levels) also deserves attention, given that generalized anxiety is the anxiety disorder with the greatest impact on academic and social functioning in adolescence (Essau et al., 2014).

The statistically significant association between female sex and social phobia (χ^2^ = 16.455, *p* = 0.002; V = 0.323)—a moderate effect size—precisely replicates the pattern documented by McLean et al. (2011) in their meta-analysis of 20 studies, in which SP prevalence in females tripled that observed in males during early adolescence. Interpreting this difference requires a multilevel framework. At the neurobiological level, functional neuroimaging studies have documented greater amygdala and anterior cingulate cortex activation in adolescent females compared to same-age males when exposed to social evaluation stimuli, activation that correlates with social anxiety scores (Guyer et al., 2008). This greater amygdala reactivity appears to be mediated by the modulatory influence of estrogens on serotonergic and GABAergic neurotransmission systems involved in the threat response (Goel & Bale, 2010). At the cognitive level, adolescent females display a greater tendency toward social rumination and elaborated processing of interpersonal rejection signals (Nolen-Hoeksema & Hilt, 2009; Hankin et al., 1998), which perpetuates and amplifies social anxiety through negative feedback cycles between cognition and emotion. At the sociocultural level, body objectification—the process by which the adolescent female internalizes the external evaluative perspective on her own body (Fredrickson & Roberts, 1997)—has been intensified by exposure to social media content promoting unattainable aesthetic standards, exacerbating body self-monitoring and public shame (Fardouly & Vartanian, 2015). The absence of statistically significant associations between sex and higher-order dimensions (total anxiety and total internalization)—despite the clinically relevant percentage differences observed—may be explained by insufficient statistical power from the sample size to detect small-to-moderate magnitude effects in variables with four response categories.

The documented internalizing comorbidity pattern—12 of 15 subdimension pairs with statistically significant associations; χ^2^ between 10.431 and 89.467—is theoretically consistent with two complementary frameworks. On one hand, the ‘p’ factor model of Caspi et al. (2014), which posits the existence of a latent general psychopathology factor that mediates the correlation between mental disorders in a manner analogous to the ‘g’ factor of general intelligence; in this model, the ‘p’ factor has its most intense expression precisely in internalizing disorders (Krueger & Markon, 2006). On the other hand, the model of intergenerational risk transmission (Silberg et al., 2010) and the development of anxiety as a process of temporal accumulation of genetic and environmental vulnerabilities (Hankin et al., 2018). The largest magnitude correlations (MDD-SA: χ^2^ = 89.467; PD-OCD: χ^2^ = 88.693) are consistent with prior studies identifying separation anxiety as a developmental precursor to major depressive disorder (Lewinsohn et al., 1998; Silberg et al., 2010) and the relationship between panic and OCD through shared mechanisms of interoceptive hyperresponsivity and attentional biases toward threat (Taylor, 1995). From an intervention standpoint, these comorbidity patterns strongly support the relevance of transdiagnostic programs (Barlow et al., 2017; Unified Protocol) over disorder-specific approaches, especially when resources are limited and the objective is to maximize population reach.

The non-significant negative correlations between age and scores on SA, MDD, and PD (ρ between −0.026 and −0.197; *p* > 0.05) are consistent with longitudinal studies observing little variation in internalizing symptomatology intensity in the 12–15 age range in the absence of stressful life events (Merikangas et al., 2010). The exception was the significant negative correlation between age and SP (r = −0.270, *p* = 0.016), suggesting a moderate reduction in social anxiety as early adolescence progresses, possibly mediated by increased social skills development, consolidation of personal identity, and reduced novelty of peer social evaluation situations (Steinberg & Morris, 2001).

Regarding the observed pre-post changes, the reductions in clinical impairment levels across all dimensions—with the largest magnitudes in SP (Δ = −25.0 pp females; Δ = −14.1 pp males) and GAD (Δ = −21.8 pp females)—are consistent with the meta-analytic findings of Neil and Christensen (2009) on school anxiety prevention interventions (average d = 0.31), and with those of Durlak et al. (2011) on SEL programs (d = 0.24 in internalizing symptoms). As a hypothesis for future research, the potential mechanisms of action of the various program components could be interpreted as follows: receptive music therapy acts by reducing sympathetic autonomic activation through heart rate modulation and galvanic skin response via auditory-limbic pathways (Thaut & Hoemberg, 2014; Gold et al., 2006), creating optimal arousal conditions for subsequent emotional learning; through heart rate modulation and galvanic skin response via auditory-limbic pathways (Thaut & Hoemberg, 2014; Gold et al., 2006), creating optimal arousal conditions for subsequent emotional learning; the cognitive–behavioral component operates through modification of attentional biases and cognitive appraisal patterns that maintain anxious cycles; the social skills and assertive communication component acts directly on the perceived social skill deficit that characterizes social phobia; and the body expression and mindfulness component reduces residual physiological activation through top-down regulation mechanisms of the autonomic nervous system (Sibinga et al., 2016; Mehling et al., 2011). The more pronounced reduction in the male group for SP (Δ = −14.1 pp in clinically significant; Δ = +27.2 pp in normal) could be explained by the statistical ceiling effect in the female group—which started from such high baseline impairment that even a substantial reduction maintained considerable residual impairment levels—but also by males’ greater receptivity to behavioral techniques of graduated exposure to evaluative social situations (Lewinsohn et al., 1998).

However, the persistence of notably higher residual impairment levels in the female group after intervention—41.7% of females at the clinically significant level of SP versus 5.9% of males—raises an equity issue in treatment response that deserves careful analysis. Garber et al. (2009) and Zahn-Waxler et al. (2008) have consistently documented that universal school interventions produce smaller effect sizes in adolescent females for social anxiety and rumination dimensions, which have led to calls for the development of gender-sensitive interventions that explicitly address the psychological mechanisms most prevalent in this group: body objectification and its cognitive correlates, normative pressure from digital media, ruminative processing, and over-relational responsibility. An intervention program that included specific modules addressing these mechanisms—for example, psychoeducation on body objectification or cognitive defusion training for ruminative thoughts—could significantly increase efficacy in the female group (McLean et al., 2011; Nolen-Hoeksema & Hilt, 2009).

### 4.1. Limitations and Future Research Directions

This study presents several limitations that must be considered when interpreting the results. The most important is the absence of a control group, which makes it impossible to rule out alternative explanations for the changes observed between T1 and T2: spontaneous maturation effects, regression to the mean, repeated testing effects, Hawthorne effects (López-Valverde Centeno, 2015), concurrent contextual changes (Shadish et al., 2002), or simply the natural maturation and progression through the school year, which may account for pre-post differences independent of the treatment. Convenience sampling, restricted to a single school in a specific neighborhood, limits external validity and the generalizability of results to other Spanish adolescent populations. The sample size (n = 79), although adequate for most planned analyses, compromises statistical power to detect small magnitude effects and renders results from the undisclosed subgroup (n = 5) practically uninterpretable statistically. The exclusive use of self-report as a data source introduces the risk of social desirability bias and common method variance (Podsakoff et al., 2003), particularly relevant in intervention program evaluations where participants may perceive expectations of improvement. The brevity of the program (five 45 min sessions) is likely insufficient to induce structural changes in cognitive schemas and emotional response patterns, which empirically validated programs address in an average of 10–16 sessions (Durlak et al., 2011). Similarly, the contextual information on gender-based dynamics at the school (Section 2.2) is based on informal staff observations rather than validated measures and should be interpreted with corresponding caution. An additional area for methodological refinement concerns intervention attendance: as the study prioritized pre-post assessment, session-by-session attendance was not individually recorded, precluding quantification of the intervention dose received by each participant and analysis of its relationship with outcomes. Future studies would benefit from systematically tracking session-level attendance as a dose variable, enabling per-protocol versus intention-to-treat comparisons. Finally, the absence of post-intervention follow-up prevents evaluation of the durability of observed changes.

Future research directions should prioritize: (a) replication of the study with cluster randomized controlled trial (RCT) designs at the class group level; (b) sample expansion through inclusion of multiple educational centers with varied socioeconomic profiles; (c) incorporation of multi-method measures complementing self-report with parent and teacher heteroreport assessments, structured diagnostic interviews (MINI-KID; Sheehan et al., 2010), and, if resources allow, biological stress markers (salivary cortisol); (d) design of 3-, 6-, and 12-month post-intervention follow-ups; and (e) systematic analysis of moderators (sex, social media use, family socioeconomic level, prior diagnosis) and mediators (changes in emotional regulation strategies, rumination, emotional self-efficacy, social skills) of program effects, through statistically rigorous change measurement models (mixed analysis of variance, latent growth curve models).

### 4.2. Clinical and Educational Implications

The results have relevant implications for school nursing practice and educational policy. First, the high prevalence of SP and GAD in this sample—even accounting for possible overestimation due to study design—suggests that school health protocols should incorporate systematic screening of internalizing symptomatology using validated instruments such as the RCADS, at least once per academic year at secondary level. Second, the descriptive reductions observed following the multicomponent program, despite the limitations of the present design, suggest that structured emotional health promotion interventions may be worth incorporating into ordinary curricular programming, as a complement—not a substitute—to available mental health care resources. Third, the school nurse emerges in this study as the ideal professional figure to coordinate and manage these interdisciplinary interventions, consistent with the functions recognized by the Consejo Andaluz de Colegios de Enfermería (2022) and with international evidence on the role of school nurses in mental health promotion (Maughan, 2003). These preliminary findings call for further evaluation of the role of school nurses in mental health promotion and support the need for broader, controlled studies to assess the potential benefits of institutionalizing the school nursing figure in Spanish public secondary education centers.

## 5. Conclusions

This study contributes to the evidence base on the emotional health profile of adolescents enrolled in socioeconomically vulnerable settings and on the feasibility of multicomponent intervention programs in ordinary school settings, with preliminary descriptive changes in symptomatology that warrant confirmation through controlled studies. Four main conclusions are derived from the results.

First, social phobia and generalized anxiety are the predominant psychopathological manifestations in this sample, with prevalences substantially exceeding normative thresholds, especially in the female group. These data reveal an intervention need that is not covered by ordinary mental health care resources in the educational context studied.

Second, sex acts as a statistically significant moderator of social phobia symptomatology χ^2^ = 16.455, *p* = 0.002, V = 0.323, with a moderate magnitude effect size. The persistent gender gap both at pretest and posttest demands the design and validation of gender-sensitive interventions that address the specific psychological mechanisms—rumination, body objectification, interpersonal sensitivity, and digital normative pressure—that mediate the greater vulnerability of adolescent girls.

Third, the strong relationship and symptom overlap between clinical subdimensions—with 12 of 15 pairs showing statistically significant associations—supports the theoretical and practical relevance of transdiagnostic approaches in the design of school emotional health interventions, as opposed to disorder-specific approaches.

Fourth, the multicomponent intervention program produced clinically relevant reductions in impairment levels across all evaluated dimensions and subdimensions in the short term, with reductions being most pronounced in social phobia and generalized anxiety. However, the absence of complete clinical remission, the persistent gender differences, and the methodological limitations inherent to the quasi-experimental design without a control group highlight the need for longer interventions, with longitudinal follow-up and evaluation through randomized controlled trials.

In summary, this work provides preliminary empirical evidence in favor of the systematic implementation of empirically based school emotional health programs, coordinated by school nursing professionals, as a public health strategy to address the growing burden of internalizing disorders in the Spanish adolescent population.

## Figures and Tables

**Table 1:** Detailed description of the five-session multicomponent emotional health intervention program.

Session	Objectives	Contents	Activities (Duration)	Resources
Session 1. Introduction to emotional health	Present the program and generate participatory motivation Establish confidentiality and group trust norms Introduce the emotional intelligence (EI) construct Administer the pretest questionnaire (RCADS)	Emotional health concept in adolescence EI model: self-awareness, self-regulation, empathy, and social skills (Goleman, 1995) Group participation expectations and norms	1. Welcome and team introduction (5 min): Brief presentation of the research team and overview of the workshop objectives to establish a safe environment. 2. ‘The question ball’ icebreaker (10 min): Participants stand in a circle and toss a soft ball. The catcher must answer a basic question about their current mood or hobbies to build group trust. 3. Participatory presentation ‘The emotional thermometer’ (15 min): Visual presentation of basic emotions. Students mark their current emotional state on a large printed “thermometer” on the board to practice emotional identification. 4. Administration of RCADS pretest (15 min): Supervised, individual completion of the paper-based questionnaire, with researchers available to clarify item meanings.	Lead researcher Support tutor Printed pretest questionnaires (RCADS) Projector and stationery
Session 2. Music therapy and emotional regulation	Use receptive and active music therapy as an emotional self-regulation tool Identify and verbalize emotional states through auditory stimuli Promote relaxation through validated music therapy techniques	Foundations of receptive and active music therapy (Bruscia, 1998) Emotional responses to rhythm, melody, and harmony Music-guided relaxation techniques (Thaut & Hoemberg, 2014)	1. Psychoeducational introduction (5 min): Brief explanation of how rhythm and melody affect heart rate, breathing, and emotional states. 2. ’Emotional musical journey’ group mural (15 min): Students listen to 3 contrasting musical tracks (1 min each) and collaboratively draw or write their emotional responses on a large shared mural using markers. 3. Music-guided progressive relaxation (10 min): Students lie on mats in a dimmed room listening to slow-tempo instrumental music, following verbal instructions to systematically tense and relax major muscle groups. 4. ‘My emotional soundtrack’ (15 min): In groups of 3–4, students discuss and list specific songs they use in their daily lives to calm down or get energized, creating a shared adaptive playlist.	Researcher Music therapist or psychologist trained in music therapy Audio playback equipment Post-its, cardboard, and markers
Session 3. Cognitive–behavioral emotional regulation	Identify and categorize basic and secondary emotions Understand the situation-cognition-emotion-behavior cycle Acquire cognitive–behavioral adaptive emotional regulation strategies	Plutchik’s (1980) model: basic emotions and the emotion wheel Cognitive-emotional cycle: situation → thought → emotion → response Regulation strategies: diaphragmatic breathing, cognitive restructuring, cognitive defusion (ACT), mindfulness	1. ‘The emotional mirror’ (8 min): In pairs, one student acts out a basic emotion using only facial expressions and posture; the partner must mirror the exact posture and guess the emotion. 2. Plutchik’s emotion wheel (12 min): Handout-based activity where students color and categorize primary and secondary emotions, learning how complex emotions are formed. 3. Role-playing ‘The emotional traffic light’ (15 min): Students act out triggering scenarios (e.g., a bad grade or a fight). They practice the steps: Red (stop and breathe), Yellow (think of consequences), Green (choose an adaptive response). 4. ‘My emotional toolbox’ (10 min): Individual worksheet where students write down 3 personal, adaptive cognitive–behavioral strategies (e.g., diaphragmatic breathing, talking to a friend) to use when overwhelmed.	Researcher Clinical or educational psychologist Emotion wheel (group and individual format) Templates for ‘toolbox’
Session 4. Assertive communication and social skills	Discriminate and practice the three styles of interpersonal communication Develop active listening and emotional validation skills Learn assertive conflict resolution techniques	Communication styles: passive, aggressive, and assertive (Alberti & Emmons, 1978) Communication components: verbal, non-verbal, and paraverbal Assertive techniques: first-person messages (‘I’ statements), broken record, fogging	1. ‘Emotional broken telephone’ (7 min): A whispered phrase about a complex emotional situation is passed around the circle to demonstrate how easily verbal communication and intentions are distorted. 2. Forum theater ‘The three styles’ (13 min): Groups of 4 are given a conflict script. They must act it out three times, demonstrating the differences and outcomes of passive, aggressive, and assertive responses. 3. ‘I messages vs. You messages’ (10 min): Pairs practice converting accusatory “You” statements (e.g., “You always ignore me”) into assertive “I” statements (e.g., “I feel sad when I am not heard”). 4. ‘The shipwreck conflict’ (15 min): A collaborative survival game where the group must negotiate limited resources, practicing active listening, emotional validation, and consensus-building without aggression.	Researcher Psychologist or social educator Cards with conflict situations Whiteboard and flipchart
Session 5. Body expression, mindfulness, and closing	Reduce anxious physiological activation through body awareness techniques Promote emotional regulation through body and mindful attention Integrate learning from previous sessions and consolidate group belonging	Psychophysiology of anxiety: stress response and autonomic nervous system Somatic mindfulness and body expression (Mehling et al., 2011; Röhricht, 2009) Progressive muscle relaxation and adapted yoga techniques	1. ‘The body traffic light’ (8 min): Physical warm-up where the instructor calls out colors (Red = freeze, Yellow = slow mindful movement, Green = fast movement) to connect external cues with physiological activation. 2. ‘Body mirror’ (15 min): In pairs, one student initiates slow, continuous body movements while the other synchronizes and mirrors them perfectly to foster somatic awareness and non-verbal empathy. 3. ‘Stretch, release, and express’ (12 min): A guided sequence of 4 basic, beginner-friendly yoga poses focusing on grounding, balance, and releasing stored muscle tension. 4. Diaphragmatic breathing + guided mindfulness (5 min): Seated exercise focusing on belly breathing (inhaling 4 s, holding 2 s, exhaling 6 s) while observing physical sensations without judgment. 5. ‘The appreciation circle’ & Posttest (12 min): Final circle where each participant shares one positive takeaway from the program, followed by the supervised completion of the RCADS posttest questionnaire.	Researcher 4th-year Nursing student (Univ. of Huelva) Support tutor Large space, mats, audio equipment

**Table 2:** Kolmogorov–Smirnov goodness-of-fit test (with Lilliefors correction) for quantitative study variables (n = 79).

Variable	D Statistic (K-S)	df	*p*-Value	Distribution
Age	0.440	78	<0.001	Non-normal
Separation Anxiety (SA)	0.165	78	<0.001	Non-normal
Panic Disorder (PD)	0.128	78	0.003	Non-normal
Social Phobia (SP)	0.091	78	0.177	Normal
Generalized Anxiety Disorder (GAD)	0.069	78	0.200	Normal
Major Depressive Disorder (MDD)	0.132	78	0.002	Non-normal
Obsessive–Compulsive Disorder (OCD)	0.149	78	<0.001	Non-normal
Total Anxiety	0.071	78	0.200	Normal
Total Internalization	0.058	78	0.200	Normal

**Table 3:** RCADS dimensions and subdimensions by sex at pretest (T1) and posttest (T2).

Dimension/Subdimension	Severity Level	Female T1 (n = 39)	Female T2 (n = 36)	Male T1 (n = 35)	Male T2 (n = 34)	Undisclosed T1 (n = 5)	Undisclosed T2 (n = 4)	Sig. Sex (T1)
Total Anxiety	Normal	48.72%	66.7% (Δ + 17.9)	77.14%	91.2% (Δ + 14.1)	80.00%	50.0%	n.s. (*p* = 0.083)
	Mild Impairment	17.95%	8.3%	11.43%	0.0%	20.00%	50.0%	
	Clinically Significant	30.77%	25.0%	11.43%	8.8%	0.00%	0.0%	
Total Internalization	Normal	61.54%	69.4% (Δ + 7.9)	85.71%	93.9% (Δ + 8.2)	60.00%	100.0%	n.s. (*p* = 0.106)
	Mild Impairment	10.26%	13.9%	8.57%	0.0%	40.00%	0.0%	
	Clinically Significant	25.64%	16.7%	5.71%	6.1%	0.00%	0.0%	
Separation Anxiety (SA)	Normal	79.49%	83.3%	85.71%	91.2%	100.00%	75.0%	n.s. (*p* = 0.468)
	Mild Impairment	17.95%	13.9%	14.29%	8.8%	0.00%	25.0%	
	Clinically Significant	2.56%	2.8%	0.00%	0.0%	0.00%	0.0%	
Generalized Anxiety Disorder (GAD)	Normal	17.95%	33.3%	40.00%	44.1%	20.00%	25.0%	n.s. (*p* = 0.091)
	Mild Impairment	35.90%	38.9%	28.57%	35.3%	80.00%	75.0%	
	Clinically Significant	38.46%	16.7% (Δ − 21.8)	25.71%	14.7% (Δ − 11.0)	0.00%	0.0%	
	Severe Impairment	7.69%	11.1%	5.71%	5.9%	0.00%	0.0%	
Major Depressive Disorder (MDD)	Normal	56.41%	58.3%	57.14%	55.9%	40.00%	25.0%	n.s. (*p* = 0.283)
	Mild Impairment	30.77%	27.8%	37.14%	38.2%	40.00%	75.0%	
	Clinically Significant	10.26%	13.9%	5.71%	2.9%	20.00%	0.0%	
	Severe Impairment	2.56%	0.0%	0.00%	2.9%	0.00%	0.0%	
Panic Disorder (PD)	Normal	53.41%	63.9% (Δ + 10.5)	65.71%	85.3% (Δ + 19.6)	100.00%	50.0%	n.s. (*p* = 0.136)
	Mild Impairment	23.08%	22.2%	28.57%	11.8%	0.00%	50.0%	
	Clinically Significant	15.38%	13.9%	5.71%	2.9%	0.00%	0.0%	
	Severe Impairment	5.13%	0.0%	0.00%	0.0%	0.00%	0.0%	
Social Phobia (SP)	Normal	12.83%	27.8% (Δ + 15.0)	25.71%	52.9% (Δ + 27.2)	20.00%	0.0%	** (*p* = 0.002)
	Mild Impairment	20.51%	22.2%	54.29%	38.2%	40.00%	50.0%	
	Clinically Significant	66.67%	41.7% (Δ − 25.0)	20.00%	5.9% (Δ − 14.1)	40.00%	50.0%	
	Severe Impairment	0.00%	8.3%	0.00%	2.9%	0.00%	0.0%	
Obsessive–Compulsive Disorder (OCD)	Normal	46.15%	66.7% (Δ + 20.6)	68.57%	70.6%	40.00%	25.0%	n.s. (*p* = 0.071)
	Mild Impairment	41.03%	22.2%	11.43%	20.6%	60.00%	75.0%	
	Clinically Significant	10.26%	11.1%	20.00%	5.9%	0.00%	0.0%	
	Severe Impairment	2.56%	0.0%	0.00%	2.9%	0.00%	0.0%	

Notes: “Sig. sex (T1)” refers to Pearson’s Chi-square test for the association between sex and each dimension at pretest (Section 3.2.1); ** *p* < 0.01; n.s. = not significant. No equivalent significance test by sex was conducted at posttest (T2) in the current dataset. Δ = change in percentage points from T1 to T2 (only reported for categories where this was calculated in the original manuscript). The “Undisclosed” columns (n = 5 at T1; n = 4 at T2) are reported for completeness and consistency with the original Table 2 and Table 3; given the very small subgroup size, these percentages should be interpreted with caution and are not amenable to statistical testing (see Section 3.1.2). SA: separation anxiety; GAD: generalized anxiety disorder; MDD: major depressive disorder; PD: panic disorder; SP: social phobia; OCD: obsessive–compulsive disorder.

**Table 4:** Statistically significant associations between pairs of RCADS subdimensions (Pearson Chi-square test). Pairs with *p* ≥ 0.05 are not displayed.

Subdimension Pair	df	Pearson χ^2^	*p*-Value	Cramer’s V	Effect Size
SA ↔ PD	6	34.572	<0.001	0.468	Large
SA ↔ SP	4	10.431	0.034	0.257	Moderate
SA ↔ OCD	6	20.058	0.003	0.356	Large
GAD ↔ PD	9	21.496	0.011	0.301	Large
GAD ↔ SP	6	23.694	0.001	0.387	Large
GAD ↔ OCD	9	21.100	0.012	0.298	Large
MDD ↔ SA	6	89.467	<0.001	0.753	Large
MDD ↔ PD	9	51.879	<0.001	0.468	Large
MDD ↔ OCD	9	20.314	0.016	0.293	Large
PD ↔ SP	6	16.186	0.013	0.320	Moderate
PD ↔ OCD	9	88.693	<0.001	0.612	Large
SP ↔ OCD	6	16.570	0.011	0.324	Moderate

df: degrees of freedom Cramer’s V calculated as V = √(χ^2^/(n·k)), where n = 79 and k = min(rows − 1, columns − 1) for each contingency table (df = 4 → k = 2; df = 6 → k = 2; df = 9 → k = 3). Effect size classified per Cohen’s (1988) conventional thresholds, adjusted for k: for k = 2, small < 0.21, moderate 0.21–0.34, large ≥ 0.35; for k = 3, small < 0.17, moderate 0.17–0.28, large ≥ 0.29. SA: separation anxiety; GAD: generalized anxiety disorder; MDD: major depressive disorder; PD: panic disorder; SP: social phobia; OCD: obsessive–compulsive disorder. All *p*-values are two-tailed.

**Table 5:** Spearman correlation matrix: separation anxiety (SA), major depressive disorder (MDD), panic disorder (PD) and age (n = 79). Values include ρ and *p*-value.

Variable	Age	SA	MDD	PD
Age	1.000	−0.026 (*p* = 0.822)	−0.050 (*p* = 0.664)	−0.197 (*p* = 0.081)
Separation Anxiety (SA)	−0.026	1.000	0.411 ** (*p* < 0.001)	0.579 ** (*p* < 0.001)
Major Depressive Disorder (MDD)	−0.050	0.411 **	1.000	0.681 ** (*p* < 0.001)
Panic Disorder (PD)	−0.197	0.579 **	0.681 **	1.000

** *p* < 0.001 (two-tailed). ρ: Spearman Rho correlation coefficient.

**Table 6:** Spearman correlation matrix: social phobia (SP), generalized anxiety (GAD), total anxiety and total internalization, with Pearson r for age correlations (n = 78–79).

Variable	SP	GAD	Total Anxiety	Total Intern.
Social Phobia (SP)	1.000	0.587 ** (*p* < 0.001)	0.830 ** (*p* < 0.001)	0.809 ** (*p* < 0.001)
GAD	0.587 **	1.000	0.760 ** (*p* < 0.001)	0.704 ** (*p* < 0.001)
Total Anxiety	0.830 **	0.760 **	1.000	0.979 ** (*p* < 0.001)
Total Internalization	0.809 **	0.704 **	0.979 **	1.000
Age (Pearson r)	−0.270 * (*p* = 0.016)	0.015 (*p* = 0.893)	−0.119 (*p* = 0.295)	−0.100 (*p* = 0.382)

** *p* < 0.001; * *p* < 0.05 (two-tailed). ρ: Spearman Rho. r: Pearson (age row only, due to normal distribution of SP, GAD, total anxiety, and total internalization).

**Table 7:** Wilcoxon signed-rank test for matched pre-post (T1 → T2) comparisons.

Dimension	n	M T1	M T2	Improved	Worsened	Tied	Z	*p*	r
Total Anxiety	74	35.89	30.45	46	24	4	−2.86	0.004 **	0.34
Total Internalization	74	44.30	38.34	48	25	1	−2.85	0.004 **	0.33
Social Phobia (SP)	74	13.45	10.80	50	19	5	−3.93	<0.001 ***	0.46
Generalized Anxiety Disorder (GAD)	74	8.14	7.24	39	27	8	−2.08	0.037 *	0.24
Obsessive–Compulsive Disorder (OCD)	74	5.30	4.53	35	21	18	−1.87	0.060	0.22
Panic Disorder (PD)	74	6.42	5.57	37	27	10	−1.45	0.144	0.17
Separation Anxiety (SA)	74	3.04	2.51	36	26	12	−1.41	0.153	0.16
Major Depressive Disorder (MDD)	74	8.49	7.91	36	28	10	−0.95	0.339	0.11

Notes: * *p* < 0.05; ** *p* < 0.01; *** *p* < 0.001. “Improved” = decrease in score from T1 to T2 (T2 < T1); “Worsened” = increase (T2 > T1). Effect size r = matched-pairs rank-biserial correlation, interpreted per Cohen (1988): small ≥ 0.10; medium ≥ 0.30; large ≥ 0.50.

## Data Availability

The data supporting the findings of this study are available upon reasonable request made to the corresponding author. Data sharing is subject to approval by the Huelva Research Ethics Committee and compliance with current data protection regulations (Regulation (EU) 2016/679).
